# Influence of stage-specific and location-related intratumoral microbiota on prognosis and metabolic reprogramming in non-small cell lung cancer

**DOI:** 10.3389/fimmu.2025.1675448

**Published:** 2026-01-05

**Authors:** Yuwei Zhao, Zhuang Tong, Lin Liu, Rongjie Yang, Zhongfeng Chen, Zheng Wang

**Affiliations:** Department of Thoracic Surgery, Cancer Hospital of China Medical University, Liaoning Cancer Hospital and Institute, Shenyang, China

**Keywords:** non-small cell lung cancer, intratumoral microbiome, immune infiltration, prognosis, 16S rRNA sequencing, immunohistochemistry

## Abstract

**Introduction:**

Mounting evidence links intratumoral microbiota to tumor progression and immune regulation, but stage-specific and location-related microbial characteristics in NSCLC remain insufficiently explored.

**Methods:**

Tumor and adjacent non-tumor tissues from 21 NSCLC patients were analyzed using 16S rRNA gene sequencing to profile the intratumoral microbiota. Functional pathway analysis, in vitro co-culture experiments (A549/HCC827 cells), and in vivo mouse models were employed to validate microbial functions.

**Results:**

Microbial composition exhibited significant heterogeneity by tumor stage (early vs. advanced) and anatomical location (upper vs. lower lobes). Bacteroides was identified as a key genus enriched in early-stage tumors and correlated with improved prognosis. Functional analysis revealed enrichment of tumor-associated microbiota in metabolic and biosynthetic pathways (e.g., carbon metabolism, amino acid biosynthesis). Further experiments demonstrated that Bacteroides significantly suppressed NSCLC cell proliferation, inhibited migration, and promoted apoptosis, with anti-tumor effects mediated by remodeling the tumor immune microenvironment.

**Discussion:**

These findings elucidate intricate associations between intratumoral microbiota, immune cell infiltration, and patient prognosis, highlighting Bacteroides as a potential prognostic biomarker and therapeutic target for NSCLC

## Introduction

Changes in the pulmonary microbiota have been implicated that can lead to various respiratory diseases (1–4). Recent evidence shows that lung cancer tissues harbor distinct microbial communities, underscoring the potential role of microflora in oncogenesis (5–9). Previously viewed as passive bystanders, these microbial communities are now recognized to actively modulate tumor biology through mechanisms involving immune regulation, epithelial barrier disruption, and metabolic reprogramming.

In gastrointestinal malignancies such as colorectal and pancreatic cancer, the influence of the microbiota on tumor initiation and progression has been well-documented. Commensal bacteria (including pathobionts) promote a chronic inflammatory microenvironment that facilitates carcinogenesis by producing genotoxins, modifying host immune pathways, and generating reactive oxygen species (ROS) (10–14). Additionally, microbial metabolites and enzymes disrupt DNA repair mechanisms, apoptosis, and epithelial renewal processes (15–18). Notably, some types of bacteria, such as *Fusobacterium nucleatum*, enhance tumor invasion and metastasis through immune evasion and pro-inflammatory signaling (19, 20).

Beyond their roles in tumorigenesis, microbiota are key determinants of therapeutic responses. In renal cell carcinoma, melanoma, and small-cell lung cancer, certain microorganisms may alter the therapeutic effect of the death protein-1 (PD-1) or corresponding immunochemical inhibitors (PD-L1) (21–23). Furthermore, bacterial enzymes degrade chemotherapeutic agents like gemcitabine, potentially contributing to drug resistance in pancreatic and lung cancers (11, 24). Overall, the results suggest that tumor-associated microorganisms not only influence malignant development, but also disease progression and treatment outcomes.

Although intratumoral bacteria were first reported over a century ago, systematic investigations into their identity, functional roles, and clinical significance have only recently begun (25, 26). In the context of lung cancer, several studies have attempted to characterize airway- or tumor-associated microbiota. For instance, Lee et al. analyzed bronchoalveolar lavage fluid and demonstrated that microbial β-diversity significantly differed between lung cancer and benign disease, although α-diversity remained comparable (27). Similarly, Zheng et al. identified low-abundance bacterial species such as *Bacteroides pyogenes* and *Burkholderia mallei* gathered in tumor tissues of NSCLC patients (28). These findings highlight the unique microbial ecosystems present within tumor environments and prompt further exploration into their biological relevance.

To date, the comprehensive characterization of the intratumoral microbiota in NSCLC, especially regarding clinical and pathological features such as tumor stage, anatomical location, and patient prognosis, remains insufficiently addressed. In this study, we systematically profiled the intratumoral microbiota using 16S rRNA gene sequencing of paired tumor and adjacent non-tumorous lung tissues from NSCLC patients. We aimed to investigate microbial variations associated with tumor progression and spatial distribution, assess their prognostic relevance, and integrate microbial functional prediction with host transcriptomic analysis to explore potential interactions between microbiota and the tumor microenvironment. These findings present novel insights into the spatial and stage-specific heterogeneity of the NSCLC microbiome and establish a foundation for identifying microbial biomarkers and developing microbiome-modulating therapeutic strategies. Such insights are anticipated to enhance precision prevention and treatment approaches, ultimately improving clinical outcomes for lung cancer patients.

## Materials and methods

### Sample acquisition and analysis in NSCLC research

In the context of this research, we scrutinized specimens from 21 individuals diagnosed with NSCLC, treated at Liaoning Cancer Hospital between July 2021 and December 2022. Tumor tissues and matched adjacent non-cancerous tissues were collected from NSCLC patients undergoing surgical resection. Tumor samples were obtained from the central region of the resected lesion, avoiding areas of necrosis. Adjacent non-cancerous tissues were defined as macroscopically normal lung parenchyma located at least 5 cm away from the tumor margin, confirmed to be free of malignant infiltration by pathological examination. All specimens were immediately snap-frozen in liquid nitrogen and stored at –80°C until subsequent analysis. A rigorous histological examination was employed to affirm the normal or neoplastic nature of each tissue sample. Alongside, a comprehensive array of clinical and pathological data was amassed, including age, gender, smoking history, disease stage, pathological types, PD-L1 status, EGFR mutation, and metastasis sites, as delineated in **Table 1**. 21 NSCLC patients were categorized into two groups: the early-stage group (Stage I–III, n=11), defined as tumors without distant metastasis (M0); and the advanced-stage group (Stage IV, n=10), defined as tumors with confirmed distant metastasis (M1a/b/c). This classification follows the common disease progression-based paradigm in lung cancer microbiome research. Adjacent non-tumorous (paracancerous) tissues were selected as internal controls because they share the same host genetic background, environmental exposures, and sampling procedures as tumor tissues. This design minimized inter-individual variability and enabled assessment of tumor-specific alterations relative to each patient’s own microenvironment.

**Table 1:** Clinical characteristics of NSCLC patients.

Characteristics	Categories	Case number	%
Total cases		21	100
Gender	Male	10	71.43
	Female	11	28.57
Smoking history	Never smoking	12	40.48
	Smoking	8	57.14
	Smoking quitted	1	2.38
Major stage	I	1	2.38
	II	2	4.76
	III	8	45.24
	IV	10	47.62
Pathological type	ADC	10	50
	SCC	7	40.48
	ASC	2	4.76
	Others	2	4.76
PD-L1 positive		5	23.81
EGFR mutation		3	21.43
Metastasis	Mediastinal lymph nodes	4	21.43
	Lung	3	21.43
	Bone	2	19.05
	Liver	2	14.29
	Brain	5	11.9
	Pleura	1	4.76
	N	Mean	SD
Age (years)	21	62.77	8.25
Cigarettes per year	21	398.77	55.72

### DNA isolation and 16S rRNA gene amplification

DNA extraction from NSCLC tumors and adjacent non-cancerous biopsy specimens were conducted by QIAamp Fast DNA Tissue Kit (Qiagen, Germany). The V4 region of the 16S ribosomal RNA (rRNA) gene was amplified by polymerase chain reaction (PCR) using universal primers: 515F (5′-GTGCCAGCMGCCGCGGTAA-3′) and 806R (5′-GGACTACHVGGGTWTCTAAT-3′), both of which were tagged with distinct barcodes. The resultant PCR products were purified using AMPure XT beads (Beckman Coulter Genomics, Danvers, MA, USA) and quantified with Qubit (Invitrogen, USA). Subsequent evaluation of the purified PCR products was carried out with Illumina library quantification kits (Kapa Biosciences, Woburn, MA, USA).

### Processing and analysis of sequencing data

The initial sequencing data were processed using Quantitative Insights Into Microbial Ecology 2 (QIIME2, version 2020.2). In brief, single-end reads were allocated to their respective samples via unique barcodes and truncated to remove the barcode and primer sequences. These demultiplexed reads underwent denoising with the q2-dada2 plugin within QIIME2, facilitating quality control, chimera detection and elimination, and the establishment of amplicon sequence variants (ASVs) and their sequences. ASVs were taxonomically classified using the SILVA database classifier (version 138).

### Cell culture and microorganism preparation

A549 and HCC827 NSCLC cell lines were used in this study. Both of them were obtained from Procella Biotechnology Co., Ltd. (Wuhan, Hubei Province, China). A549 cells (CL-0016, Procell, China) were maintained in Ham’s F-12K medium, which was supplemented with 10% fetal bovine serum (FBS) and 1% penicillin-streptomycin (P/S) (CM-0016, Procell, China). HCC827 cells (CL-0094, Procell, China) were cultured in RPMI-1640 medium containing 10% FBS and 1% P/S (CM-0094, Procell, China). All cell cultures were placed at 37°C in a humidified incubator with 5% CO_2_. 0.25% Trypsin-EDTA (PB180229, Procell, China) were used to digestive cells, and reseeded for subsequent experiments. *Bacteroides fragilis* strain (WN-JF65661) was purchased from WARNER Biological Technology Co., Ltd. (Wuhan, China). The strain identity was confirmed by 16S rRNA gene sequencing prior to experiments. *Bacteroides* was cultured in *Bacteroides* Phage Recovery Medium Broth (BPRMB) under strictly anaerobic conditions at 37°C to ensure an ideal growth environment. After reaching the desired growth phase, *Bacteroides* cultures were harvested, and centrifuged. The resulting cell pellet was rinsed softly with sterile phosphate-buffered saline (PBS) to eliminate media constituents. The bacteria were resuspended in the culture medium at varying concentrations to prepare inoculum doses of 10² CFU/mL (low concentration) and 10^4^ CFU/mL (high concentration) for co-culture experiments with A549 or HCC827 cells.

### Co-culture experiment

Both A549 and HCC827 cells were seeded at a density of 5 × 10^4^ cells per well in 96-well plates and incubated at 37°C with 5% CO_2_ for 24 hours to allow cell adhesion. Afterward, the cells were treated with *Bacteroides* at concentrations of 10² CFU/mL or 10^4^ CFU/mL and incubated for an additional 48 hours. Cell proliferation, apoptosis, metastasis, etc., were then assessed to evaluate the effects of *Bacteroides* exposure.

Cell proliferation ability was measured via the Cell Counting Kit-8 (CCK-8) assay (BS350B, Biosharp, China). 48 hours of *Bacteroides* treatment afterwards, 10 µL CCK-8 solution was added to the cell well, the mixture was put into the 37°C incubator for 2 hours. Absorbance was then measured at 450 nm with the microplate reader, which quantitatively measures cell viability (29, 30).

The Transwell assay was performed using Transwell chambers equipped with 8 µm pore-size polycarbonate membrane inserts. For seeding, the upper chamber received A549 or HCC827 cells (2×10^5^ cells per well) suspended in serum-free medium (either Ham’s F-12K or RPMI-1640). The lower chamber was loaded with complete growth medium, which consisted of Ham’s F-12K or RPMI-1640 supplemented with 10% FBS. *Bacteroides fragile* suspension was added to the upper cavity and co-cultured with the cells. The Transwell plates were then incubated at 37°C in a 5% CO_2_ environment for 24 to 48 hours. After incubation, cells were fixed with 4% paraformaldehyde (P0099, Beyotime, China) for 20 minutes and then stained with 0.1% crystal violet (G1064, Solarbio, China) for 15 minutes. Migratory or invasive cells were finally quantified microscopically to assess their capacity (31).

The EdU assay was carried out to analyze cell proliferation. Cells were incubated with EdU Proliferation Assay Kit (ab219801, Abcam, UK) following the manufacturer’s protocol. After an incubation period of 2–4 hours, the cells were fixed in fixative solution and incubated for 15 minutes and then permeabilized with a permeabilization buffer and treatment for 20 minutes. Subsequently, a click reaction was performed to label the Edu-incorporated DNA. The cells were then counterstained with a nuclear stain DAPI (C1006, Beyotime, China) to visualize the nuclei. The Edu-positive cells were detected and quantified using fluorescence microscopy to determine the proportion of proliferating cells.

Apoptotic cells were detected by the Tunel assay. Cells were first fixed with 4% paraformaldehyde for 30 minutes and then washed softly once with PBS. The Immunostaining Strong Permeabilization Solution (P0097, Beyotime, China) was added and the cells were incubated for 5 minutes at room temperature. Following two washes with PBS, cells were treated with 50 μl of TUNEL Detection Solution (C1086, Beyotime, China) according to the manufacturer’s instructions and incubated at 37°C in the dark enviroment for 60 minutes. After three washes with PBS, the apoptotic cells with positive Tunel staining were detected and quantified using fluorescence microscopy to assess the apoptosis rate of the cells (32).

Following harvesting, cells were stained with Annexin V-fluorescein isothiocyanate (FITC) and propidium iodide (PI) (C1062S, Beyotime, China) prior to flow cytometric analysis. This staining allows for the differentiation between different states cells. The proportion of apoptotic cells in each treatment group was quantitatively determined by flow cytometry.

### *In vivo* experiment

Six- to eight-week-old male C57BL/6 mice were purchased from Beijing Vital River Laboratory Animal Technology Co., Ltd. and acclimated for one week under specific pathogen-free (SPF) conditions with ad libitum access to food and water. All animal experiments were approved by the Ethics Committee of Laboratory Animals, Liaoning University of Traditional Chinese Medicine (No. CMUKT2024075).

To establish NSCLC tumor models, A549 cells were resuspended in sterile PBS to a density of 1 × 10^6^ cells per 100 µL, followed by subcutaneous injection into the right flank of each mouse. Approximately one week post-injection, when tumors reached palpable size, mice were randomly assigned to three groups: (i) Control group (mice injected with PBS only, without tumor cell implantation), (ii) NSCLC group (tumor-bearing mice established by subcutaneous injection of A549 cells, without additional treatment), and (iii) Bacteroides group (tumor-bearing mice established by A549 cells, followed by treatment with Bacteroides fragilis). The primary comparison was made between the NSCLC and Bacteroides groups to assess the microbial effect, while the PBS-only control group served as a baseline reference. Mice were monitored daily for body weight, tumor volume, and general condition. The endpoint criteria included a maximum tumor diameter of 1.5 cm, rapid ulceration or necrosis, or >15% body weight loss. At the endpoint, animals were humanely euthanized by CO_2_ inhalation followed by cervical dislocation, in accordance with institutional guidelines. Blood was collected by cardiac puncture immediately after euthanasia, and serum was separated by centrifugation at 3,000 rpm for 10 minutes at 4°C and stored at –80°C until analysis. Tumors were excised, weighed, and processed for histology, immunohistochemistry, and molecular assays.

At the endpoint, mice were euthanized, and tumor tissues were excised for further analysis. Tumor tissues were fixed in 10% formalin, embedded in paraffin, sectioned, and stained with antibodies against PD-1 and CD163 to evaluate immune cell infiltration and expression. Additionally, antibodies against Ki-67 and Caspase-3 were used to observe the dynamic changes of tumor cells. The sections were colored with diaminobenzidine (DAB), and images were captured by an optical microscope for the analysis of the expression levels of the above markers.

According to the manufacturer, the amount of inflammatory cytokines in the serum, including tumor-labeled embryonal antigens (CEA), TNF-α, and IL-10, are detected by Enzyme-linked immunosorbent assay (ELISA) kits. The absorbance of each maker at specified wavelengths was determined using a porous plate reader and the concentration was calculated according to a standard curve.

To analyze protein expression, RIPA inhibitors contain protease (P0013B, Beyotime, China) that breaks down cells or tissues. All proteins were quantified (P0006, Beyotime, China) and then transmitted to the PVDF cell membranes. In order to detect apoptosis-related proteins, the experiment focused on anticells associated with these proteins. Subsequent steps involved incubating membranes with primary antibodies targeting key apoptotic markers such as Bcl-2 (HY-P80029, MedChemExpress, China, 1:1000 dilution) and Caspase-3 (HY-P80046, MedChemExpress, China; 1:1000 dilution) at 4°C overnight. For proteins related to cell proliferation, we used the primary antibodies which were against CDK-4 (12790, Cell Signaling Technology (CST), USA, 1:1000 dilution), CDK-6 (3136, CST, USA, 1:1000 dilution), and Cyclin D1 (2978, CST, USA, 1:1000 dilution). In the case of metastasis-related proteins, we selected the primary antibodies against TWIST1 (90445, CST, USA, 1:1000 dilution), TWIST2 (ab66031, Abcam, UK, 1:1000 dilution), N-Cadherin (14215, CST, USA, 1:1000 dilution), and E-Cadherin (3195, CST, USA, 1:1000 dilution). Followed by incubation with HRP-conjugated secondary antibodies (ab6702, Abcam, UK, 1:3000 dilution). Signals were visualized using an ECL chemiluminescent substrate, with relative protein expression levels quantified using Image J analysis software.

### Bioinformatics analysis and statistical methodology

The microbial sequence data were presented in FASTQ format. Raw sequences were processed using vsearch for sequence dereplication and feature table construction, and denoised with usearch. Taxonomic annotation was performed against the RDP database (version 18), while a parallel BLAST-based annotation using the SILVA 138 database was also conducted to ensure robustness. A typical operational taxonomic unit array (OTU) is taxonomically assigned through BLAST using the SILVA 138 database. Differential abundance of taxonomic features between each group was assessed via Linear Discriminant Analysis Effect Size (LEfSe), and the Kruskal-Wallis rank sum test. The functional spectrum was predicted via the community phylogenetic survey method based on 16S rRNA gene OTUs.

β-diversity was calculated using Bray–Curtis distance (the most widely used metric for microbial community comparison). Group differences in β-diversity were tested via PERMANOVA (permutational multivariate analysis of variance) with 999 permutations, and corresponding R² and P-values were reported. For direct comparisons between tumor and adjacent non - tumorous tissues, analyses were stratified by tumor stage (early/advanced) and anatomical location (upper/lower lobe), with α-diversity (Shannon index) compared via two-tailed t-test and β-diversity via PERMANOVA.

GO enrichment analysis was executed using the ‘ClusterProfiler’ package in R. To account for multiple testing, P-values were adjusted using the Benjamini–Hochberg false discovery rate (FDR) method, and pathways with FDR-adjusted P < 0.05 were considered significantly enriched. Gene set enrichment analysis (GSEA) was also conducted with ‘ClusterProfiler’ using the same adjustment criteria. Pathway activity within TCGA samples was computed using the GSVA package, and correlation analyses were conducted with the ‘ggcor’ package.

Differences in Optimal cut-off values and survival among groups were determined using the R ‘survminer’ package. Variance in microflora expression across groups was calculated with the ‘rstatix’ package, while the ‘ggplot2’ package facilitated the computation of bacterial positive rate disparities and boxplot generation. Correlations between bacterial species were assessed using the ‘ggcorrplot’ package, which also produced a heatmap.

### Statistical analyses

Data are presented as mean ± standard deviation (SD). All statistical analyses and data visualization were performed using R software (version 4.0.3). Continuous variables were compared using the Wilcoxon rank-sum test, while categorical variables were analyzed using the Chi-squared test or Fisher’s exact test as appropriate. Beta diversity differences (Bray–Curtis distance) between groups were evaluated by permutational multivariate analysis of variance (PERMANOVA). Intra-group agreement of categorical variables was assessed using Cohen’s kappa statistic. For functional enrichment and pathway analyses, P-values were adjusted for multiple testing using the Benjamini–Hochberg false discovery rate (FDR) method, with FDR-adjusted P < 0.05 considered statistically significant. Unless otherwise specified, a two-tailed P < 0.05 was regarded as the threshold for statistical significance.

## Results

### Intratumoral microbiome heterogeneity and clinical correlations in NSCLC

**Figure 1:**
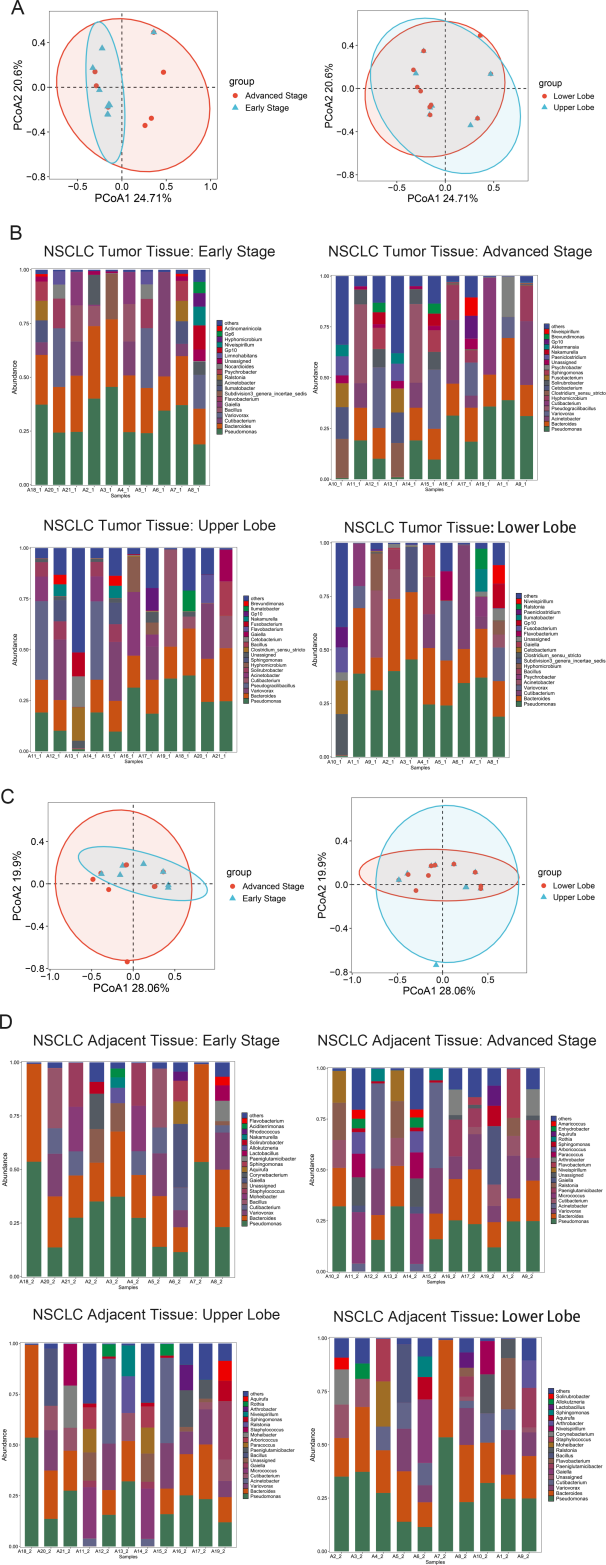
Varied β-diversity in intratumoral and adjacent microbiota across NSCLC stages and locations. **(A)** Beta diversity of the intratumoral microbiome, stratified by stage (early or advanced) and locations (upper or lower). **(B)** Fluctuations in the prevalence of the twenty predominant genus-level microbes, categorized by stage (early or advanced) and locations (upper or lower). **(C)** Beta diversity of the microbiome adjacent to the cancer, differentiated by stage (early or advanced) and locations (upper or lower). **(D)** Variations in the abundance of the twenty most prevalent genus-level microbes, dissected by stage (early or advanced) and locations (upper or lower). All β-diversity analyses used Bray–Curtis distance; PERMANOVA p-values for group separations are reported in the main text. Stratified direct comparisons (tumor vs. paracancerous by stage/location) are based on α-diversity (Shannon index) and β-diversity (Bray–Curtis distance) with statistical significance verified by t-test (α-diversity) and PERMANOVA (β-diversity).

Using 16S rRNA sequencing, we profiled the intratumoral microbiome of 21 NSCLC tumor samples and found significant heterogeneity associated with tumor stage and lung location. β-diversity analyses (based on Bray–Curtis distance) revealed clear separations between early- and late-stage tumors (PERMANOVA, R²=0.16, P = 0.008) and between upper and lower lobes (PERMANOVA, R²=0.14, P = 0.012) in intratumoral samples (**Figure 1A**). For paracancerous samples, β-diversity also differed significantly by stage (PERMANOVA, R²=0.15, P = 0.010) and location (PERMANOVA, R²=0.13, P = 0.015; **Figure 1C**). To identify dominant genera, we combined relative abundance quantification (**Figures 1B, D**) with statistical validation: In tumor tissues, Bacteroides (mean relative abundance: 8.2 ± 1.5%) and Pseudomonas (mean relative abundance: 7.6 ± 1.3%) ranked among the top 3 most abundant genera; in adjacent non - tumorous tissues, their mean relative abundances were 7.9 ± 1.2% and 7.1 ± 1.4%, respectively (top 4 genera). Further, LEfSe analysis (LDA score > 3.0, P<0.05) confirmed these two genera as consistently enriched taxa across both tissue types, supporting their classification as dominant genera.

### Interplay between tumor stage, microbiota, and lung cancer survival

**Figure 2:**
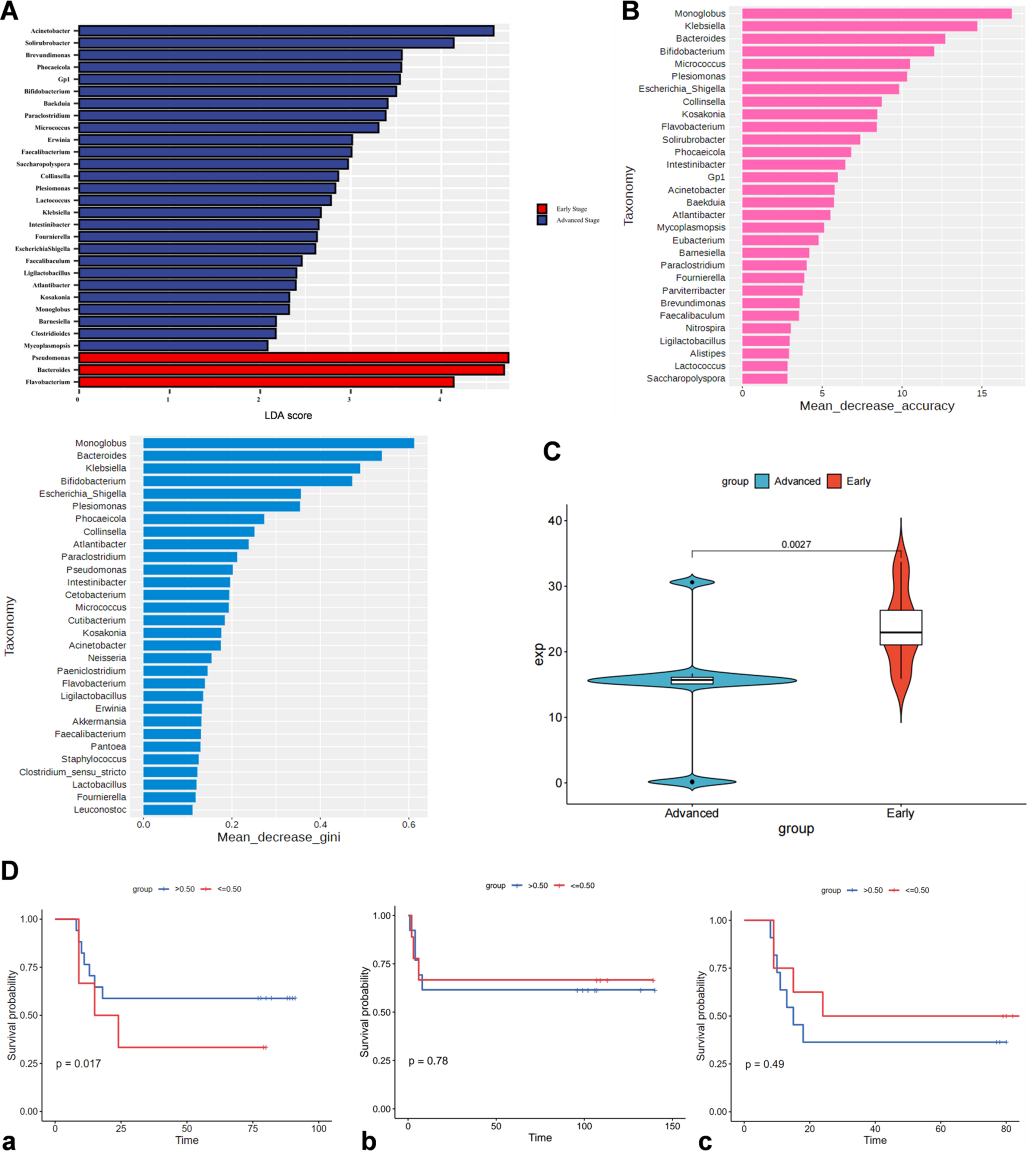
Elucidation of stage-associated microflora in lung cancer. **(A)** LEfSe analysis of the 16S rRNA gene sequencing data, adjusted by MaAsLin2, comparing early and advanced stage cancers. **(B)** The top 30 bacteria delineated between early and advanced stages, evaluated by mean decrease accuracy and mean decrease Gini in a random forest model. **(C)** The differential abundance of *Bacteroides* genera in early (blue) versus advanced (red) stages. **(D)** Kaplan–Meier plots for *Bacteroides***(a)**, *Acinetobacter***(b)**, and *Solirubrobacter***(c)** with >50%/<50% abundance cutoff (chosen based on median abundance, see main text for details). All plots use consistent colors: red = early-stage, blue = advanced-stage.

To exclude histological subtypes as a confounder for inter-stage intratumoral microbial differences, we analyzed subtype distribution (**Table 1**): no significant ADC/SCC/ASC proportion differences between early- (I–III) and advanced-stage (IV) groups (P = 0.68). Subtype-stratified β-diversity (Bray–Curtis) showed significant inter-stage microbial differences within ADC and SCC, but no inter-subtype differences within the same stage, confirming inter-stage microbial differences reflect disease progression, not subtypes. To further explore the clinical significance of intratumoral microbial heterogeneity, we identified stage-associated microbial signatures and assessed their prognostic value in NSCLC. A random forest classifier (based on Mean Decrease Accuracy and Mean Decrease Gini; **Figure 2B**) pinpointed key genera for stage discrimination, with *Monoglobus* and *Bacteroides* as top contributors. Complementary LEfSe analysis (**Figure 2A**) revealed distinct profiles: *Pseudomonas* and *Bacteroides* were enriched in early-stage tumors (red), while *Acinetobacter* and *Solirubrobacter* dominated late-stage lesions (blue)—indicating a stage-dependent ecological shift. Boxplot analysis confirmed higher *Bacteroides* abundance in early-stage samples (P = 0.0027; **Figure 2C**), supporting its role as an early-disease microbial biomarker. Although Bacteroides was also detected in adjacent non - tumorous tissues, its enrichment specifically in early-stage tumors and its positive association with survival suggest that it may serve as a context-dependent biomarker of early disease progression rather than a tumor-exclusive signal. Kaplan–Meier analysis showed high *Bacteroides* abundance (>0.50 relative abundance) correlated with improved overall survival (P = 0.017; **Figure 2D**), whereas late-stage-enriched genera (e.g., *Acinetobacter*, *Solirubrobacter*) had no prognostic association (**Figure 2D**). The >50% vs. <50% cutoff for Bacteroides abundance was chosen based on the median relative abundance of Bacteroides across all samples (median = 0.48%), a common approach in microbial prognostic analyses to balance group sizes and maximize prognostic relevance. These findings position *Bacteroides* as both a stage-discriminating taxon and potential prognostic indicator in NSCLC.

### Interplay between tumor location, microbiota, and lung cancer survival

**Figure 3:**
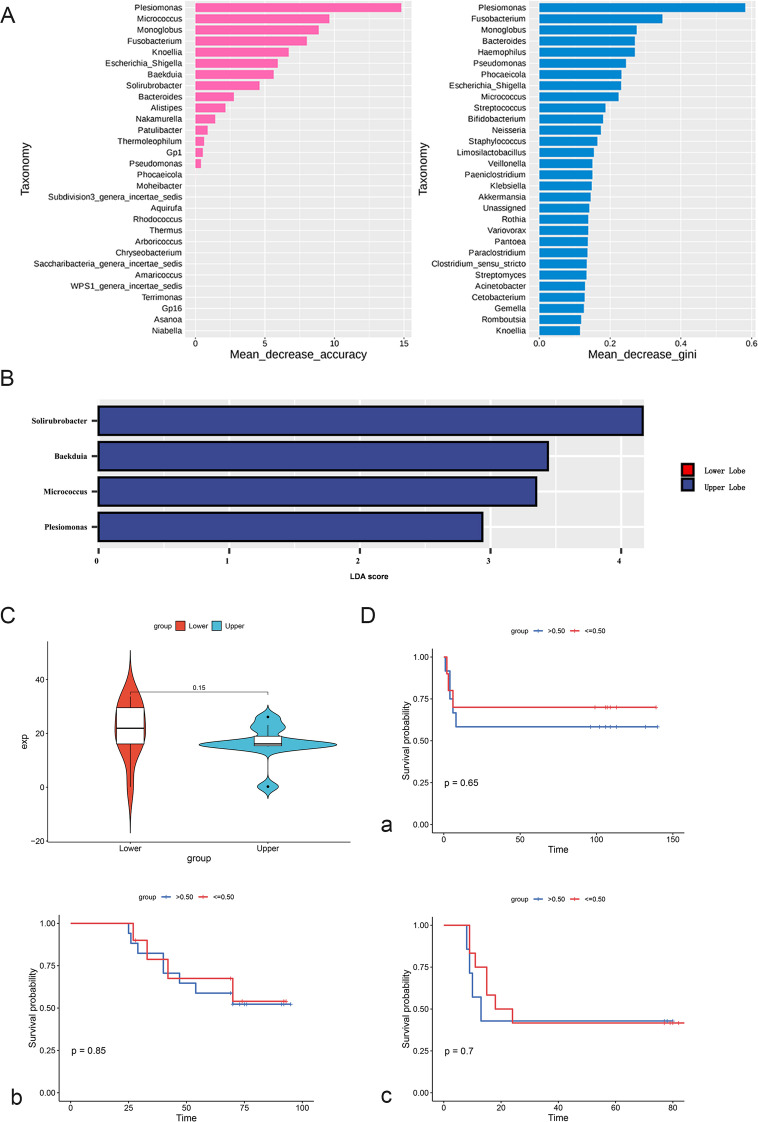
Elucidation of location-associated microflora in lung cancer. **(A)** Top 30 genera contributing to upper/lower lobe discrimination via Random Forest (Mean Decrease Accuracy and Mean Decrease Gini). **(B)** LEfSe analysis (x-axis: LDA score) showing candidate taxa enriched in upper (blue) and lower (red) lobes; MaAsLin2 was used for subsequent covariate adjustment and p-value correction. **(C)** Wilcoxon test-based boxplot of *Micrococcus* relative abundance (labeled) in upper (blue) vs. lower (red) lobes. **(D)** Kaplan–Meier plots for Plesiomonas, Solirubrobacter, and Micrococcus (each labeled) with 50% abundance cutoff (chosen based on taxon-specific median abundance, see main text for details).

To further investigate the spatial heterogeneity of the intratumoral microbiome in NSCLC, we focused on microbial signatures associated with tumor location (upper vs. lower lobe). Random forest modeling was employed to identify key genera discriminating tumor locations based on two importance metrics: Mean Decrease Accuracy and Mean Decrease Gini. As shown in **Figure 3A**, Plesiomonas, Micrococcus, and *Monoglobus* were identified as top contributors by Mean Decrease Accuracy (left panel, pink), while *Plesiomonas*, *Fusobacterium*, and *Monoglobus* ranked highest by Mean Decrease Gini (right panel, blue), with *Plesiomonas* consistently emerging as the most influential genus across both criteria. We next analyzed the abundance of representative genera in tumors located in the upper versus lower lung lobes. **Figure 3B** shows the distribution of *Solirubrobacter*, *Baekduia*, *Micrococcus*, and *Plesiomonas*. While all four genera were detectable in upper lobe tumors (blue), *Solirubrobacter* exhibited the highest abundance, whereas *Plesiomonas* was relatively less enriched, suggesting possible spatial niche preference. To validate these findings, we further compared microbial expression levels between lobes. As shown in **Figure 3C**, expression (exp) levels of *Micrococcus*—a representative location-associated genus—were significantly higher in lower lobe tumors (red) contrasted to upper lobe tumors (blue), with a mean difference of 0.15. This pattern supports the notion that specific microbial taxa may be preferentially enriched in distinct anatomical regions of the lung. Finally, Kaplan–Meier survival analysis was performed to evaluate the prognostic relevance of selected location-associated genera. As shown in **Figure 3D**, no significant survival differences were observed between high- and low-abundance groups for *Plesiomonas*, *Solirubrobacter*, or *Micrococcus*, suggesting that while these genera are spatially informative, their prognostic value may be limited. Together, these results outline a clear screening–validation–correlation framework for identifying tumor location–associated microbiota in NSCLC. Key taxa such as *Plesiomonas*, *Solirubrobacter*, and *Micrococcus* demonstrate spatial specificity, offering new perspectives for regionally tailored microbiome-based diagnostics or interventions.

### Functional pathway analysis of stage - and location - associated microbiota in NSCLC

**Figure 4:**
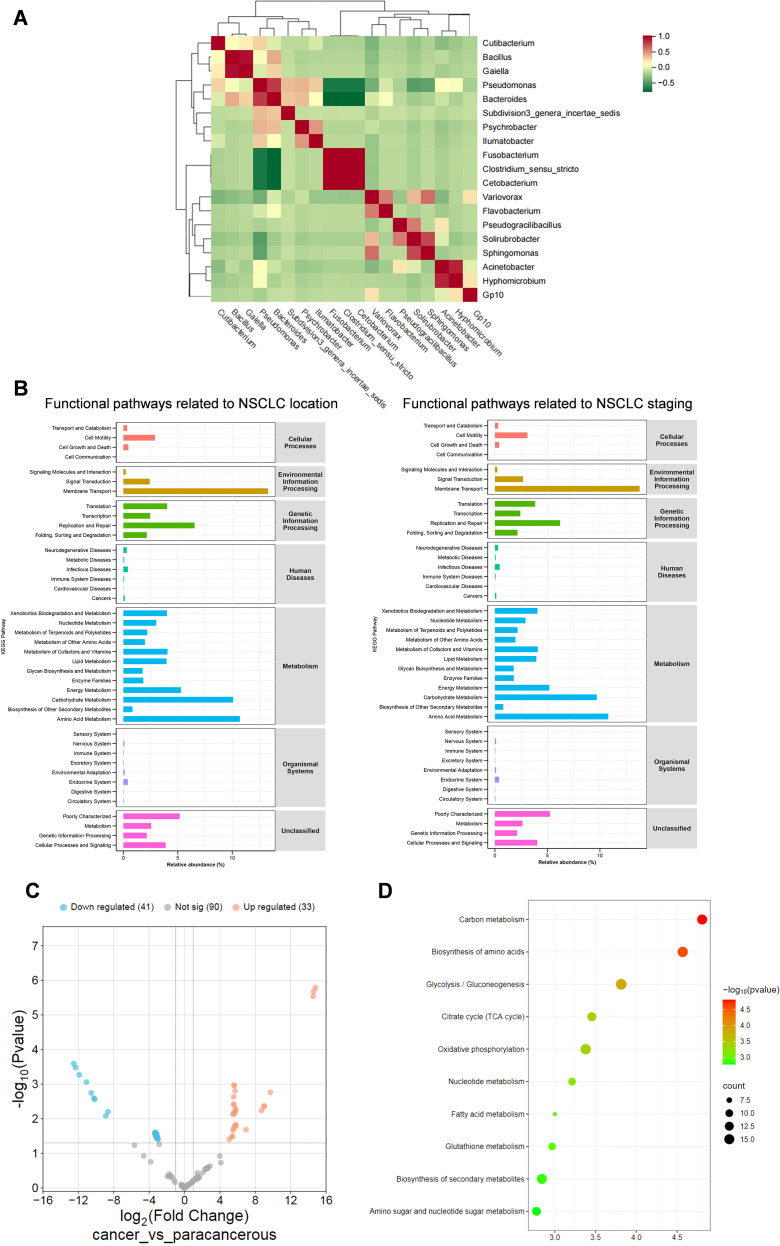
Analysis of biological processes and pathways based on stage- and location-specific genes. **(A)** Correlation assessment of intratumoral bacteria identified through a random forest model utilizing 16S rRNA sequences. Correlations were calculated using Spearman’s correlation coefficients; |r| < 0.3 was considered weak, 0.3 ≤ |r| < 0.6 moderate, and |r| ≥ 0.6 strong. P-values were adjusted using the Benjamini–Hochberg method, with significance set at p < 0.05. **(B)** Predictive insights into functional pathways associated with bacteria clusters, stratified by stage and location, as inferred by PICRUSt analysis. **(C)** Differential expression of biological pathways between tumor and adjacent non-tumorous tissues in NSCLC samples derived from TCGA LUAD (517 tumor, 59 normal) and LUSC (502 tumor, 49 normal) cohorts (accession phs000178). **(D)** Bubble plot of top 10 metabolic pathways implicated in tumor energy homeostasis and biosynthesis.

To further investigate the functional implications of microbiota associated with NSCLC tumor stage and anatomical location, we performed an integrated analysis of microbial interactions and predicted functional pathways. A genus-level co-occurrence network revealed distinct microbial interaction patterns, with genera such as *Cubobacterium*, *Bacillus*, and *Pseudomonas* displaying strong positive correlations, suggesting potential cooperative behavior within the tumor microenvironment, while negative correlations indicated possible microbial competition(**Figure 4A**). Functional prediction analysis showed that microbiota associated with tumor location were enriched in pathways related to “Transport and Catabolism” and “Signaling Molecules and Interaction,” whereas stage-associated microbiota demonstrated differential enrichment in similar categories, including “Translation,” indicating that microbial functions shift with disease progression and tumor positioning(**Figure 4B**). Moreover, transcriptome-based comparison of tumor and adjacent tissues from TCGA data identified 33 upregulated and 41 downregulated pathways, with significant enrichment in metabolic processes such as “Carbon Metabolism” and “Biosynthesis of Amino Acids,” implicating microbiota-driven alterations in core metabolic and biosynthetic functions (**Figures 4C, D**). Collectively, these analyses revealed that stage- and location-specific microbial communities in NSCLC exhibit distinct co-occurrence patterns and functional profiles, particularly enriched in metabolic and biosynthetic pathways such as carbon metabolism and amino acid biosynthesis. Notably, these functional pathways were closely associated with tumor progression and regional heterogeneity, suggesting that intratumoral microbiota may actively shape tumor microenvironment via metabolic regulation.

### *In vitro* and *in vivo* verification of microbiota-immune environment interaction

To further verify the biological relevance of these microbiota–host interactions, we performed some experiments to investigate the functional impact of *Bacteroides*—a representative microbial genus identified in the tumor-associated microbiota—on NSCLC cell behavior and immune modulation.

**Figure 5:**
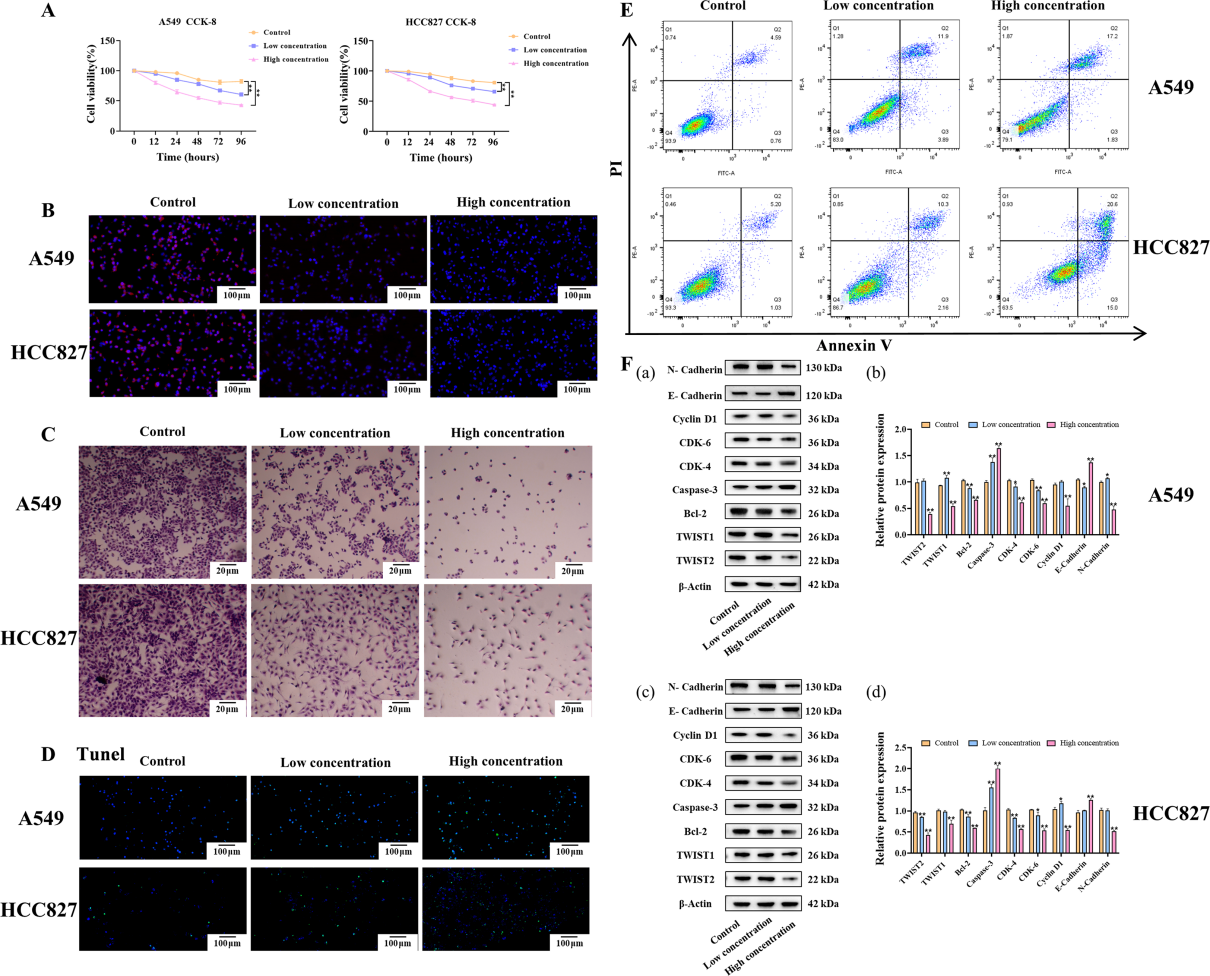
Effects of different concentrations of *Bacteroides* on A549 and HCC298 cells. **(A, B)** CCK-8 and EdU assay for cell proliferation in A549 and HCC827 cells after co-culture with *Bacteroides* at low (10² CFU/mL) or high (10^4^ CFU/mL) concentrations; **(C)** The Transwell assay assessed cell migration capacity; **(D, E)** TUNEL and Annexin V/PI double staining flow cytometry were used to detect cell apoptosis. **(F)** Western blot detection of apoptosis proteins Bcl-2 and Caspase-3, proliferation - related proteins CDK-4, CDK-6, Cyclin D1, and metastasis - related proteins TWIST1, TWIST2, N-Cadherin, E-Cadherin and quantification of Western blot results using Image J.

In the co-culture experiment, the CCK-8 method detection showed that different concentrations of *Bacteroides* had significant inhibitory effects on the proliferation of NSCLC cell lines (p < 0.05), and the inhibitory effect was more obvious in the high-concentration group, with the most significant effect at 48 hours. The low-concentration group also had inhibition but to a weaker degree. The cell viability of the control group decreased only slightly (**Figure 5A**). EdU experiments further confirmed that the DNA synthesis activity of cells was reduced and cell proliferation was inhibited after treatment with *Bacteroides* (**Figure 5B**).

The Transwell assay results showed that the migration ability of A549 and HCC827 cells revealed significant inhibition of cell migration in the high concentration treatment groups compared to the control groups (p < 0.01). In both cell lines, the number of migrated cells was markedly reduced in the high concentration group, with a more moderate but still notable reduction observed in the low concentration group (p < 0.05) (**Figure 5C**). The control groups exhibited robust cell migration, suggesting that *Bacteroides fragile* may have an inhibitory effect on the metastasis of cancer cells.

The TUNEL method detection showed that the apoptosis rate of the *Bacteroides fragile* treatment group was increased (p < 0.01), indicating that it could promote apoptosis (**Figure 5D**). Annexin V/PI flow cytometry also showed that the apoptosis rate were consistent with the TUNEL method (**Figure 5E**).

Additionally, Western blot analysis showed that the expression of Bcl-2 was decreased in the *Bacteroides fragile* treatment group, while the expression of Caspase-3 was increased (p < 0.01), further supporting the pro-apoptotic effect of the microorganism on NSCLC cells (**Figure 5F**). In addition, it was found that proteins related to cell proliferation like CDK-4, CDK-6 and Cyclin D1, were also decreased in the flimsy *Bacteroides* treatment group (p < 0.01), further enhancing its ability to inhibit the proliferation of NSCLC cells. At the same time, the expression of transfer-related proteins were also detected. It was found that TWIST1 and TWIST2 transcription factors expressed decreased (p < 0.01), N-Cadherin expressed decreased (p < 0.01) and E-Cadherin expressed increased (p < 0.01) significantly in the *Bacteroides fragile* treatment group. This suggests that the microbe may reduce cell metastasis by inhibiting the epithelial-mesenchymal transition (EMT) process. These results indicate that *Bacteroides* has a significant inhibitory influence on NSCLC cells via different mechanisms, including promoting apoptosis, decreasing cell proliferation and reducing cell metastasis.

**Figure 6:**
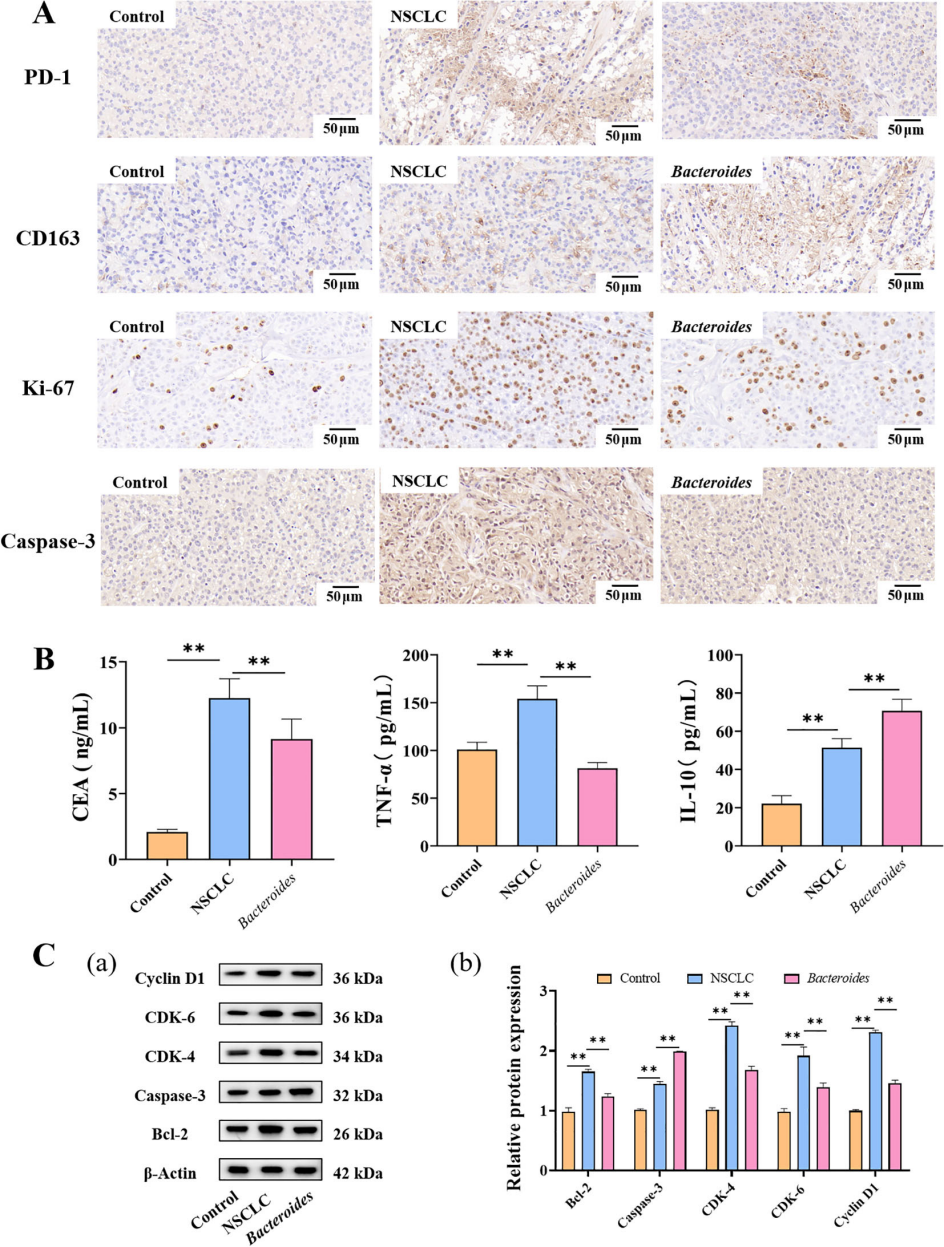
Verification of the effects of *Bacteroides* on NSCLC in C57BL/6 mouse models. **(A)** Detection of key tumor marker CEA and inflammatory factors TNF-α and IL-10 expression levels using ELISA kits; **(B)** Illustrative immunohistochemistry (IHC) images of samples labeled with antibodies against PD-1, CD163, Ki-67, and Caspase 3. **(C)** Western blot detection of apoptotic proteins Bcl-2 and Caspase-3, as well as proliferation-related proteins CDK-4, CDK-6, Cyclin D1 and quantification of Western blot results using Image J. ** denotes a highly significant statistical difference (p < 0.01) between compared groups.

In the *in vivo* experiment, to evaluate the role of *Bacteroides fragile* on NSCLC, a mouse xenograft model was established and the differences between the control group, the NSCLC cell injection group, and the microbial treatment group were compared. Immunohistochemical analysis revealed that PD-1 expression in tumor tissues of the microbial treatment group was significantly higher than that in the control group (p < 0.05), whereas CD163 expression was significantly decreased (p < 0.05). This suggests that *Bacteroides fragile* may influence tumor development by adjusting the immunosuppressive factors levels in the tumor microenvironment. (**Figure 6A**). Further immunohistochemical detection revealed that the proliferation marker Ki-67 was decreased in the microbial treatment group (p < 0.01), whereas the expression of apoptosis marker Caspase-3 was increased (p < 0.01) (**Figure 6A**). These observations indicate that *Bacteroides* has the effect of inhibiting the proliferation of tumor cells and promoting their apoptosis.

Biochemical analysis of mouse blood samples showed that the levels of tumor marker CEA and inflammatory factor TNF-α were increased in the microbial treatment group (p < 0.01), while IL-10 (an anti-inflammatory factor) was decreased (p < 0.01) (**Figure 6B**). These data suggest that *Bacteroides fragile* treatment may activate the immune response in mice, thereby affecting the growth of NSCLC and the remodeling of the immune environment.

In addition, apoptotic proteins Bcl-2 and Caspase-3, along with proliferation-related proteins CDK-4, CDK-6, and Cyclin D1, were quantitatively measured by Western blotting (**Figure 6C**). The results demonstrated that Bcl-2 was decreased, whereas Caspase-3 was increased (p < 0.01) in the microbial treatment group, which further supported its promoting effect on apoptosis. At the same time, the expressions of CDK-4, CDK-6, and Cyclin D1 were decreased (p < 0.01) in the microbial treatment group, indicating that *Bacteroides* could effectively inhibit cell proliferation.

Collectively, these combined results suggest that *Bacteroides* significantly inhibits the growth of NSCLC through multiple mechanism. These findings not only deepen our understanding of microbiome-immune environment interactionst but also provide new perspectives for developing microbial-based cancer treatment strategies.

Notably, Bacteroides emerged as a key taxon across multiple analyses: It was identified as a dominant genus in both tumor and adjacent non - tumorous tissues (via relative abundance quantification and LEfSe), showed stage-specific enrichment (higher in early-stage tumors, validated by Wilcoxon test and Random Forest), and correlated with improved prognosis (Kaplan–Meier analysis). This cross-validation across distinct analytical approaches highlights Bacteroides as a potentially important microbial marker in NSCLC. However, its dual role—supporting host homeostasis in adjacent non - tumorous tissues while potentially influencing tumor progression in cancerous tissues—warrants further investigation, as the underlying mechanisms (e.g., strain-specific effects, metabolic crosstalk with host cells) remain to be clarified. Future studies focusing on isolate-level functional experiments could help dissect the specific contributions of Bacteroides to NSCLC progression.

## Discussion

This study comprehensively analyzed and highlighted the stage-specific and histotype-related characteristics of the intratumoral microbiota in NSCLC and their impact on prognosis and the immune landscape. The findings offering novel insights for future research and clinical applications.

The significant heterogeneity in microbial composition based on tumor stage and anatomical location is consistent with previous research indicating that the intratumoral microbiota can influence tumor development and progression (33). For instance, the enrichment of *Bacteroides* in early-stage tumors and its association with better prognosis align with studies showing that certain microbial genera can modulate the immune response and affect patient outcomes (34). This dualistic role of *Bacteroides*, as both a homeostasis-supporting genus in adjacent non - tumorous tissues and a potential contributor to immune suppression or tumor progression in tumor tissues, highlights the tissue-context dependency of microbial functions (35).

The identification of stage-associated microbial signatures, such as *Bacteroides*, as potential prognostic indicators in NSCLC, is supported by evidence from other cancer types. For example, in esophageal squamous cell carcinoma (ESCC), higher levels of *Fusobacterium nucleatum (Fn)* showed an association with advanced tumor and reduced survival rates (36). Similarly, in primary liver cancer, the prognosis of patients positively showed a correlation with the increase in the context of the relative abundance of *Pseudomonas* (37). These findings suggest that intratumoral microbiota can serve as valuable biomarkers for cancer prognosis.

The functional pathway analysis revealing enrichment in metabolic and biosynthetic pathways, such as carbon metabolism and amino acid biosynthesis, is in line with studies indicating that intratumoral microbiota can drive alterations in core metabolic functions (38). This metabolic regulation by microbiota may actively shape the tumor microenvironment, influencing tumor progression and regional heterogeneity (39). However, it should be noted that the functional inference is predictive and derived from 16S rRNA sequencing (e.g., PICRUSt), rather than from direct metagenomic or metatranscriptomic measurements. This approach may introduce bias and may not fully reflect the true functional capacity of the microbial community. Future studies using shotgun metagenomics or metatranscriptomics are warranted to validate and refine these functional predictions.

The *in vitro* and *in vivo* experiments demonstrating the inhibitory effects of *Bacteroides* on NSCLC cell proliferation, migration, and apoptosis, as well as its ability to modulate the immune environment, provide biological plausibility for the observed associations. Similar findings have been reported in other cancer types, where specific bacteria can influence tumor growth and immune responses. For example, intratumoral *Bifidobacterium* can trigger the activation of dendritic cell through the STING signaling pathway, enhancing antitumor immunity (40, 41). These experimental results support the potential therapeutic application of microbiota in cancer treatment.

However, there are several limitations that warrant consideration. First, the cohort of 21 patients is relatively small for robust subgroup analyses. This may limit the generalizability of the findings and the statistical power of certain comparisons. These promising results therefore warrant future validation in larger, independent, and preferably multi-center cohorts. Second, the use of 16S rRNA sequencing limits the resolution to genus level and does not provide direct information on microbial function. Therefore, references to Bacteroides fragilis in our experimental validation represent the selected strain used in co-culture and *in vivo* assays, rather than species-level identifications derived from sequencing data. Additionally, the predictive functional analysis applied in this study may not accurately capture microbial activities; integrating metagenomic or metatranscriptomic approaches in future research will be crucial. Moreover, this study did not include a comprehensive analysis of the tumor immune microenvironment. Data on tumor-infiltrating lymphocytes, MDSCs, other immune cell subsets, or correlations between Bacteroides abundance, PD-L1 expression, neoantigen load, and immune infiltration were not assessed, which represents an important limitation that will be addressed in future work through integrated immunophenotyping and spatial analyses (42). Third, the experiments focused mainly on *Bacteroides* additional functional studies are needed to evaluate the effects of other key microbial taxa in NSCLC (43).

In conclusion, this study reveals distinct stage- and location-related differences in the intratumoral microbiota of NSCLC and highlights their potential roles in tumor progression, immune modulation, and prognosis. While encouraging, these findings should be interpreted with caution given the limited sample size, and further studies are required to validate and extend these results. These findings provide a basis for further research on the diagnosis and treatment of microlung cancer.

## Conclusion

This study systematically elucidates the heterogeneity of the intratumoral microbiome in NSCLC and its association with tumor stage, location, and patient prognosis. We identify *Bacteroides* as a key microbial genus enriched in early-stage tumors and linked to favorable survival outcomes. Functional pathway analysis and experimental verification demonstrate that *Bacteroides* can regulate the tumor immune microenvironment and exert significant anti-tumor effects. These findings advance our understanding of the complex interactions between the tumor microbiome and host immunity in NSCLC, highlighting the potential of microbial components as biomarkers and therapeutic targets. Future research integrating microbial, immune, and molecular data may lay the foundation for personalized microbiome-based interventions, ultimately enhancing precision oncology for lung cancer patients.

## Data Availability

The datasets presented in this study can be found in online repositories. The names of the repository/repositories and accession number(s) can be found below: Raw data link: https://www.jianguoyun.com/p/DSIX2f4Q17jHDRi_84kGIAA.
